# Ultrahigh Nitrogen Content Carbon Nanosheets for High Stable Sodium Metal Anodes

**DOI:** 10.1002/advs.202206845

**Published:** 2023-02-15

**Authors:** Bicheng Huang, Shixiong Sun, Jing Wan, Wen Zhang, Siying Liu, Jingwen Zhang, Feiyang Yan, Yi Liu, Jia Xu, Fangyuan Cheng, Yue Xu, Yaqing Lin, Chun Fang, Jiantao Han, Yunhui Huang

**Affiliations:** ^1^ State Key Laboratory of Material Processing and Die & Mould Technology School of Materials Science and Engineering Huazhong University of Science and Technology Wuhan 430074 P. R. China; ^2^ Department of Applied Physics Chongqing University Chongqing 401331 China

**Keywords:** anode‐free, carbon nanosheets, dendrites‐free, Na metal batteries, ultrahigh nitrogen content

## Abstract

Sodium metal, with a high theoretical specific capacity of 1165 mAh g^−1^, is the ultimate anode for sodium batteries, yet how to deal with the inhomogeneous and dendritic sodium deposition and the infinite relative dimension change of sodium metal anodes during sodium depositing/stripping is still challenging. Here, a facile fabricated sodiuphilic 2D N‐doped carbon nanosheets (N‐CSs) are proposed as sodium host material for sodium metal batteries (SMBs) to prevent dendrite formation and eliminate volume change during cycling. Revealing from combined in situ characterization analyses and theoretical simulations, the high nitrogen content and porous nanoscale interlayer gaps of the 2D N‐CSs can not only concede dendrite‐free sodium stripping/depositing but also accommodate the infinite relative dimension change. Furthermore, N‐CSs can be easily process into N‐CSs/Cu electrode via traditional commercial battery electrode coating equipment that pave the way for large‐scale industrial applications. On account of the abundant nucleation sites and sufficient deposition space, N‐CSs/Cu electrodes demonstrate a superior cycle stability of more than 1500 h at a current density of 2 mA cm^−2^ with a high coulomb efficiency of more than 99.9% and ultralow nucleation overpotential, which enable reversible and dendrites‐free SMBs and shed light on further development of SMBs with even higher performance.

## Graphical abstract

A kind of ultrahigh nitrogen content carbon nanosheets is used as Na metal anode, which can be easily and continuously process into N‐CSs/Cu electrode via traditional commercial battery electrode coating equipment. It sheds light on further development of sodium metal batteries(SMBs)with even higher performance.

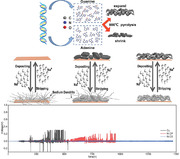

## 1 Introduction

With the rapid development of energy storage, the demand for lithium resources is increasing. However, the reserves (0.0017 wt% in crust) and uneven distribution (accounts for 6% of the world) of lithium resources have become important problems restricting the application of lithium ion batteries (LIBs) in the field energy storage. Sodium ion batteries (SIBs) may become the successor of LIBs in some fields due to the abundant sodium resources (2.3 wt% in crust) and the similar energy storage mechanism to LIBs. Among various anode materials, metallic sodium anode will be the ultimate choice for sodium battery to achieve high‐energy‐density battery systems owing to its high theoretical capacity (1165 mA h g^−1^) and a lowest operating potential (−2.714 V vs SHE).^[^ ^1^, ^2^, ^3^, ^4^ ^]^ Nevertheless, originating from sodium “hostless” nature, the infinite relative dimension change during Na plating/stripping, accompanied by the formation of Na dendrite and “dead” sodium in the anode, leading to rapid capacity degradation and potential shorting hazards in sodium metal batteries (SMBs).^[^ ^5^, ^6^, ^7^, ^8^, ^9^ ^]^ Moreover, the large infinite relative dimension change of Na metal during cycling also results in the interfacial stress between the electrodes and the separator that lead to SMBs bulge and structural failure induces. To circumvent this bottleneck, continuous effort has been made in the past years. Although artificial Na host skeletons, as previously reported, with well‐designed 3D structures and devisable chemically modified surfaces, have been proven to have clear advantages to solve the above problem due to that the well‐designed 3D structures can accommodate sodium deposition within their porous matrix to eliminate the infinite relative dimension change, simultaneously, the devisable chemically modified surfaces can guide the uniform nucleation of sodium on the host skeleton to suppress dendrite formation, but, from the perspective of industrialization, because of their costly and complex manufacturing process, most of those 3D structural Na host skeletons are too delicate to be grafted into the commercial battery.^[^ ^8^, ^10^, ^11^, ^12^ ^]^ Indeed, in the industry, practicality has been recognized as the vital assessment criterion for electrode materials. But in scientific research, until now, the industrialization of Na host skeletons for SMBs has been neglected. Fortunately, recently Hu group reported that using carbon coated aluminum foil, an industrially prepared C–Al foil, as anodes for sodium metal batteries and demonstrated that different structural carbon have distinct sodium dendrite protection performance. Although this work still does not solve the problem of volume change of Na metal anode, but provides a solution to address the dilemma that Na host skeletons cannot be industrialized.^[^ ^12^ ^]^

In this work, 2D carbon nanosheets (N‐CSs) with high nitrogen content, derived from guanine, were first employed as Na host skeletons for sodium metal batteries. Owing to the crimped and easy stacked 2D structure N‐CSs provide ample space to eliminate the volume fluctuation during sodium depositing/stripping. Additionally, in the light of density functional theory (DFT) calculation, the high N doping contents of N‐CSs also facilitate to reduce the nucleation overpotential and local current density which plays a vital role in suppressing sodium dendrite growth and diminishing dead sodium production. Most importantly, N‐CSs can be easily and continuously process into N‐CS/Cu electrode via traditional commercial battery electrode coating equipment that pave the way for large‐scale industrial applications. In actual testing, N‐CSs/Cu electrode demonstrates an superior cycle stability of more than 1500 h at a current density of 2 mA cm^−2^ with a high coulomb efficiency of more than 99.9% and an ultralow nucleation overpotential.

## 2 Results and Discussion

### 2.1 Morphology and Structure Characterization of N‐CSs and N‐CPs

**Figure 1:**
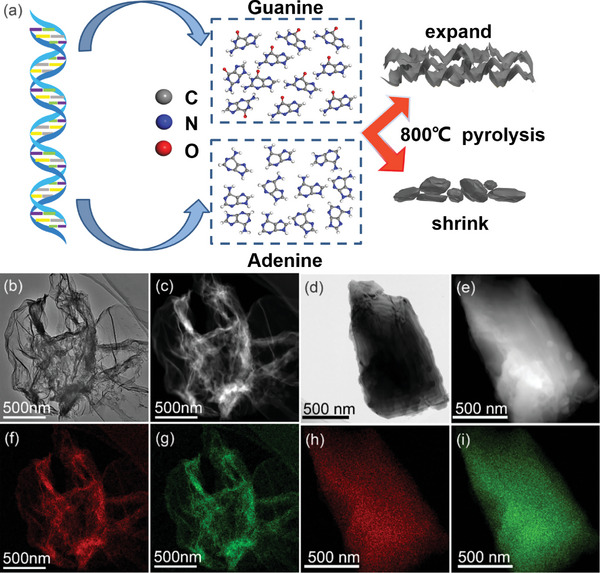
a) Schematic diagram of material synthesis. b,d) TEM image of N‐CSs and N‐CPs. c,e) High‐angle annular dark‐field (HAADF) image of N‐CSs and N‐CPs. f–i) TEM‐EDS mapping images (red‐C, green‐N) of N‐CSs and N‐CPs

Guanine, with a layered crystal structure,^[^ ^13^, ^14^ ^]^ is widely present in the biomass and is considered to be an ideal precursor for 2D carbon materials. During the pyrolysis process, the nitrogen atoms in guanine can be in situ doped into the 2D carbon nanosheets (N‐CSs) and endow N‐CSs unique characteristics. Adenine has a similar chemical composition but a completely different structural pyrolytic carbon to guanine because of the different crystal structure between guanine and adenine (**Figure** **1**a). The bulk structural pyrolytic carbon (N‐CPs) derived from adenine has a similar nitrogen content to N‐CSs and is chosen as a comparison in this work (Figure S1, Supporting Information). The detailed synthesis process of N‐CSs and N‐CPs are shown in the Experimental Section. The morphology of N‐CSs and N‐CPs were characterized by scanning electron microscopy (SEM) and transmission electron microscopy (TEM). As shown in Figure 1b,c, Figure S2a,b (Supporting Information), the curled N‐CSs derived from guanine stack with each other to form a porous structure that could provide sufficient deposition space for sodium metal. On the other side of the coin, the bulk N‐CPs evolved from adenine with irregular shape scattering in granular form. The powder size of N‐CPs is approximately distributed between 5 and 20 µm (Figure 1d,e; Figure S2d,e, Supporting Information). The energy‐dispersive X‐ray spectroscopy (EDS) mappings of the N‐CSs and N‐CPs are displayed in Figure 1f–i, and exhibit a similar element distribution, respectively. The specific elemental composition ratio obtained from TEM‐EDS analysis is shown in Table S2 (Supporting Information), N‐CSs and N‐CPs exhibit similar N content of 27.03% and 26.63%, respectively.

The specific surface area (SSA) and pore size distribution (PSD) of N‐CSs and N‐CPs were further investigated by N_2_ adsorption‐desorption isotherms and the corresponding curves and results were shown in Figure S4 and Table S1 (Supporting Information), respectively. N‐CSs exhibit significantly higher SSA (76.54 m^2^ g^−1^) and pore volume (PV) (0.2006 cm^3^ g^−1^) than that of N‐CPs (4.476 m^2^ g^−1^ and 0.0043 cm^3^ g^−1^). Combining the results of SEM and TEM, it suggests that the curled and ultrathin 2D structure endows N‐CSs with a higher specific surface area and larger pore volume than block structural N‐CPs. Besides, the curled and ultrathin 2D structural N‐CSs also exhibit a lower apparent density than block structural N‐CPs. As shown in Figure S2c (Supporting Information), at the same weight of 200 mg, the volume of N‐CSs is almost five times than that of N‐CPs. All of the above structural characteristics make N‐CSs more suitable as Na host framework material for SMBs.

The structural features of N‐CSs and N‐CPs were further studied by X‐ray diffraction (XRD) and Raman spectroscopy. As shown in Figure S5a (Supporting Information), N‐CSs and N‐CPs exhibit a similar XRD patterns with two broad peaks near 2*θ* = 26° (002 plane) and 43°(101 plane). According to the Bragg diffraction formula, the (002) face interlayer spacing of N‐CSs and N‐CPs is about 0.337 nm that are much smaller than that of biomass carbons (0.37–0.42 nm) but closely to that of graphite (0.335 nm), which is mainly caused by the N‐induced shrinkage of the carbon layer derived from the ultrahigh N content of N‐CSs and N‐CPs.^[^ ^15^, ^16^ ^]^ Given the graphite‐like interlayer spacing, N‐CSs and N‐CPs are not suitable as anode materials for NIB, but, thanks to the ultrahigh N content that provides abundant natriophilic sites, N‐CSs and N‐CPs are very suitable as Na host framework material for SMBs.^[^ ^17^, ^18^ ^]^ Raman spectra of N‐CSs and N‐CPs are shown in Figure S5b–d (Supporting Information), both samples exhibit two distinguished peaks at 1350 and 1581cm^−1^ that corresponds to D band (related to the defects and disorder in the hexagonal lattice) and G band (the vibration of sp^2^ hybridized C atoms in the 2D hexagonal lattice) of carbon materials respectively. The intensity ratio of D band and G band (I_D_/I_G_) is a commonly index that is usually used to evaluate the disorder degree of carbon materials. The I_D_/I_G_ of N‐CSs and N‐CPs is 3.19 and 3.21, respectively, and implying a similar disordered carbon structure due to the ultrahigh content N doping in the hexagonal lattice. The high content of N doping in the carbon ring introduced a lot of defects. The defects are more conducive to the initial nucleation of sodium metal, so this disordered carbon structure increases the sodium affinity sites, promoting the uniform nucleation of sodium metal in the deposition process.

**Figure 2:**
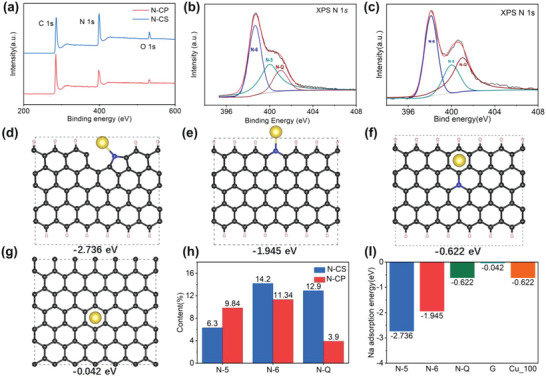
a) XPS surveys of N‐CS and N‐CP. b) N 1s XPS spectra of N‐CP. c) N 1s XPS spectra of N‐CS. Computational models of d) N‐5, e) N‐6, f) N‐Q, and g) graphene. h) Contents of N‐5, N‐6, N‐Q in N‐CS and N‐CP. i) Comparison of binding energy between different structural models and Na.

The element contents of N‐CSs and N‐CPs were further confirmed by X‐ray photoelectron spectroscopy (XPS). As shown in **Figure** **2**a, the significant N 1s peaks in the XPS survey spectra of N‐CSs and N‐CPs indicate the extremely high N contents of both samples. The element contents of N‐CSs and N‐CPs are listed in Table S3 (Supporting Information), the exactly N contents of N‐CSs and N‐CPs are 33.4 at% and 25.08 at%, respectively, which is consistent with the TEM‐EDS results. In addition, the local bonding environments of N atoms in N‐CSs and N‐CPs is further determined in the high‐resolution N 1s spectra. As shown in Figure 2b,c, the three peaks at 398.1, 400.0, and 401.0 eV in the high‐resolution N 1s spectra are attributed to pyridine‐N (N‐5), pyrrole‐N (N‐6), and graphite‐N (N‐Q), respectively. The fitting contents of N‐5, N‐6, and N‐Q in N‐CSs and N‐CPs are shown in Table S3 (Supporting Information) and Figure 2h.

It has been generally acknowledged that Na metal nucleation/plating tends to form the largest low‐energy sodium/substrate interface. From the perspective of kinetics and thermodynamics, sodium metal is more apt to nucleate and deposit in the N‐doping carbon interface. In order to verify the contribution of N atoms in different chemical environments to the sodium affinity, we further utilized density functional theory (DFT) calculations to investigate the interaction between Na and N‐doping carbon interface. The monolayer graphene with hydrogen saturation width of 14.76 Å is used as the carbon substrate model for calculation. Then the C atom is replaced with N atoms at different positions in carbon substrate to simulate the N doping in different chemical environments. As shown in Figure 2d–f, The binding energy of N‐5, N‐6, and N‐Q doped carbon substrate with Na are −2.736, −1.945, and −0.622 eV, respectively. As a comparison, the binding energy of carbon substrate and Cu (100) crystal with Na are −0.042 and −0.622 eV, respectively (Figure 2g; Figure S6a, Supporting Information). The calculated binding energy of Na with N‐5 and N‐6 doped carbon substrate is much lower than that of N‐Q doped carbon substrate and Cu (100) crystal, indicating the Na nucleation process on N‐CSs and N‐CPs is thermodynamically favorable. Furthermore, by comparing the binding energy value, N‐5, N‐6, and N‐Q doping can all reduce the binding energy of carbon substrate with Na. Among them, N‐5 doped carbon substrate has the most negative binding energy, indicating that it is easy to combine with Na. This is because the doping of N‐5 will bring more dangling bonds and introduce the most defects. N‐Q has the least defects, resulting in less increase in sodium affinity (Figure 2i). Furthermore, we calculated the binding energy of N‐5, N‐6, and N‐Q doped graphene with Na is −2.632 eV, and also has very good sodium affinity (Figure S6b, Supporting Information). On the basis of graphene doped with a single N‐6 atom, the binding energy with Na can be reduced from −1.945 to −2.44 eV by introducing another N‐6 atom. Therefore, it is not difficult to conclude that C materials with higher N content should have better sodium affinity (Figure S6c,d, Supporting Information).

### 2.2 Electrochemical Performance of N‐CSs/Cu and N‐CPs/Cu

**Figure 3:**
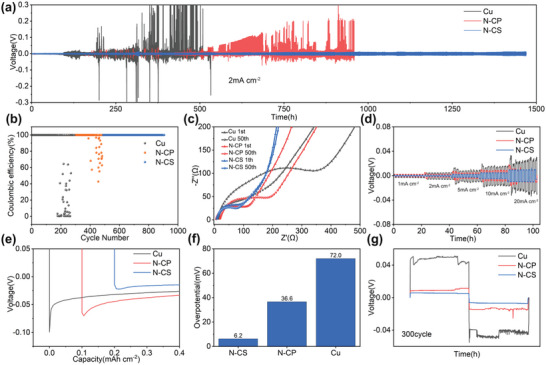
Electrochemical performance of symmetric cells with Na@N‐CSs, Na@N‐CPs, and Na@Cu. a) Cycling stability at 2 mA cm^−2^. b) Coulombic efficiency at 2 mA cm^−2^. c) Electrochemical impedance spectroscopy (EIS) at 2 mA cm^−2^. d) Rate performance at 1, 2, 5, 10, 20 mA cm^−2^. e) Nucleation voltage curve at 1 mA cm^−2^ at the 1st cycle. f) Nucleation overpotential at 1 mA cm^−2^ at the 1st cycle. g) Voltage curve at 1 mA cm^−2^ at the 300th cycle.

To further elucidate the plating and stripping of N‐CSs and N‐CPs on long‐term deep Na cycling, we fabricated the N‐CSs/Cu and N‐CPs/Cu composite electrode by utilizing commercial electrode preparation methods to coat N‐CSs or N‐CPs on copper foil (Figure S2f, Supporting Information). The compared cycling stability performance and coulombic efficiency (CE) of Na||N‐CSs/Cu, Na||N‐CPs/Cu, and Na||Cu at a current density of 2 mA cm^−2^ and an areal capacity of 2 mA h cm^−2^ are displayed in **Figure** **3**a,b, respectively. It can be seen that Na||N‐CSs/Cu maintains the ultra‐low voltage hysteresis during 1500 h ultralong cycles with the plating/stripping CE of 99.99%, but the CE of Na||N‐CPs/Cu and Na||Cu quickly drop to below 50% after 400 and 200 cycles, respectively. To explore the source for this phenomenon, the electrochemical impedance profiles of Na||N‐CSs/Cu, Na||N‐CPs/Cu, and Na||Cu after 1st and 50th cycles are investigated and given in Figure 3c. The cell impedance of Na||N‐CSs/Cu exhibits the smallest initial interfacial impedance and the negligible impedance increase after 50 cycles. This might be due to that the high N doping content of N‐CSs provide abundant sodium‐philic nucleation sites and sufficient sodium metal deposition location to reduce the local current density and inhibit the generation of dendrites. By contrast, after 50 cycles, the interface impedance of Na||N‐CPs/Cu and Na||Cu increase significantly that may originate from the growth of sodium dendrites and the formation of dead sodium after cycling. Although N‐CPs has a similar N doping amount to N‐CSs that can provides plentiful natriophilic sites beneficial to the nucleation of sodium metal, the smaller SSA of the bulk structural N‐CPs cannot provide enough space to accommodate the deposited sodium metal, which leads to the generation of sodium dendrites and the dead sodium in the process of multiple plating/stripping.^[^ ^19^, ^20^, ^21^ ^]^ The rate performance of Na||N‐CSs/Cu, Na||N‐CPs/Cu, and Na||Cu batteries were further tested at the current density of 1, 2, 5, 10, 20 mA cm^−2^, respectively. As displayed in Figure 3d, with the current density increasing, the voltage curve of Na||N‐CSs/Cu is still very stable, showing the best performance. More surprisingly, as exhibited in Figure S7 (Supporting Information), at a high current density of 10 mA cm^−2^, Na||N‐CSs/Cu still maintained a long‐term stable cycle of 500 h with no significant increase in voltage hysteresis. Compared with other recently published natriophilic substrate for sodium metal anode, the performance of N‐CSs/Cu has been very prominent.^[^ ^22^, ^23^, ^24^ ^]^ This profit from the reduction of the nucleation overpotential of sodium metal by the natriophilic N atoms, and the abundant interval space derived from the stacked 2D wrinkled N‐CSs provide sufficient sodium deposition space. The results of voltage capacity curves of Na nucleation and plating process also prove this conclusion. Figure 3e shows the voltage capacity curve of Na nucleation and plating process on N‐CSs/Cu, N‐CPs/Cu, and bare Cu substrates at a current density of 1 mA cm^−2^ and an areal capacity of 1 mA h cm^−2^. Thanks to the high N content of N‐CSs/Cu and N‐CPs/Cu to reduce the nucleation energy, the nucleation potentials of Na||N‐CSs/Cu, Na||N‐CPs/Cu were 6.2, 36.6 mV, respectively, which are much lower than that of Na||Cu (72.0 mV). It is worth mentioning that N‐CSs exhibit significantly higher SSA (76.54 m^2^ g^−1^) than that of N‐CPs (4.476 m^2^ g^−1^). At the same deposition current, the higher specific surface area is conducive to the uniform distribution of Na^+^ flux, thus reducing the local current density. Therefore, Na||N‐CPs/Cu has a higher local current density, resulting in a greater nucleation overpotential. The voltage profiles of Na||N‐CSs/Cu, Na||N‐CPs/Cu, and Na||Cu batteries at the 300th plating/stripping cycle were investigated and displayed in Figure 3g. It can be seen that at the 300th plating/stripping process, only Na||N‐CSs/Cu can keep the voltage hysteresis basically unchanged and the voltage curve smooth, indicating that the current density is relatively uniform during the plating/stripping process, and there is no obvious dendrite and dead sodium generation.

### 2.3 Analysis of Electrodes After Charge–Discharge Cycle

**Figure 4:**
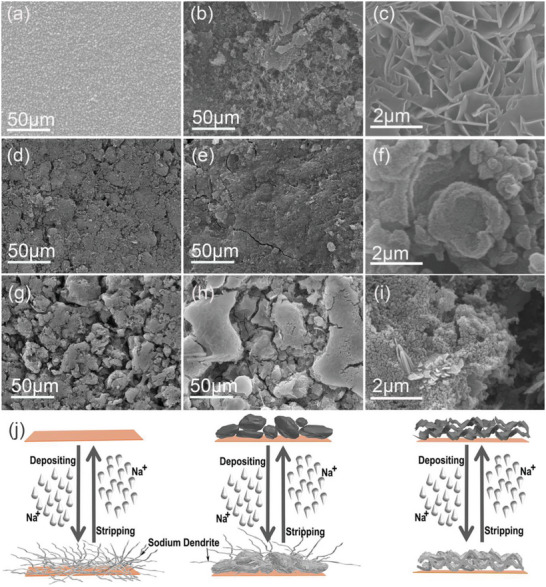
Top‐view SEM images of a–c) bare Cu, d–f) N‐CSs, g–i) N‐CPs. Among them, a,d,g) before plating, b,e,h) after plating, c,f,i) high magnification after plating. j) Schematic diagram of sodium deposition.

SEM images in **Figure** **4** exhibit the morphological evolution of Cu, N‐CSs/Cu, and N‐CSs/Cu electrodes from fresh to 300th Na plating with the areal capacities of 2 mA h cm^−2^ at the current density of 2 mA cm^−2^. By comparing the structural changes of three electrodes, it can be seen that after 300 cycles of plating/stripping, the electrodes slides were removed in the plating state. Comparing the optical photographs of the two electrodes, silvery white metal is clearly observed on the Na@N‐CPs electrode (Figure S8a,b, Supporting Information). Then the Na deposition morphology on bare Cu, N‐CSs, and N‐CPs after 300 plating/stripping cycles were further compared by top‐view and section view SEM image analysis. Figure 4b,e,h is the top view of bare Cu foil, N‐CSs, N‐CPs substrates, respectively (Figure 4b,e,h). After 300 times of plating/stripping, disordered dendritic crystals were formed on the surface of Cu foil. In the continuous plating/stripping process, crystals with weak connection may break off to form dead sodium(Figure 4b,c).^[^ ^8^ ^]^ For N‐CSs and N‐CPs, they can strongly attract sodium ions and guide the uniform nucleation of Na metal, so that the whole surface of the host structure can be used for Na plating. The layered crimp stacking structure of N‐CSs sheet increases the specific surface area. Na metal gradually fills the interlamellar voids during deposition. Therefore, N‐CSs is still very flat after deposition of Na metal (Figure 4e,f). However, N‐CPs become more aggregated and exhibit irregular features after platting, such as islands, pores, etc. (Figure 4h,i). The irregular block structure does not provide internal sodium storage space. Sodium metal is preferentially deposited on the surface of block material, and the irregular shape of block material leads to irregular deposition of Na. By comparing the high magnification SEM images before and after the deposition of Na, we can see the form of Na deposition more intuitively. When Na metal is deposited on N‐CSs substrate, it can evenly fill the space between layers, thus forming a dense plating layer (Figure S9a,b, Supporting Information).For N‐CPs, it can be clearly seen from the graph that Na metal deposited on the surface of N‐CPs in the form of inclusion, thus the volume expansion is obvious during the deposition process (Figure S9c,d, Supporting Information). To further verify the conclusion, the cross section was characterized by SEM. Compared to N‐CSs, obvious cracks were observed in the cross section of N‐CPs substrate. This is because the stress caused by expansion makes the material crack during multiple deposition/stripping processes (Figure S10a,b,d,e, Supporting Information). From the high magnification SEM images, it can also be seen that Na deposition on N‐CS substrate is uniform and smooth (Figure S10c, Supporting Information). The N‐CP substrate is deposited on the surface of the block, resulting in many holes, which eventually leads to the loose and porous deposition of Na metal (Figure S10f, Supporting Information).

**Figure 5:**
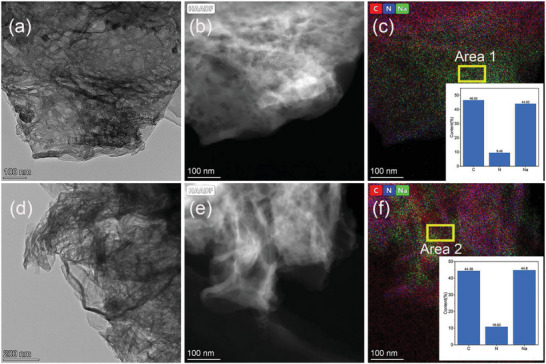
Structural characterization of plated N‐CSs and N‐CPs. a) TEM image, b) High‐angle annular dark‐field (HAADF) image, c) TEM‐EDS mapping images of Na@N‐CSs at the 1st plating d) TEM image, e) High‐angle annular dark‐field (HAADF) image, f) TEM‐EDS mapping images of Na@N‐CSs at the 300th plating (red‐C, blue‐N, green‐Na). The inset are regional element content analysis.

To further verify the excellent sodiophilicity of N‐CSs/Cu, we performed TEM‐EDS mapping images of Na@N‐CSs/Cu at different plating cycles. As shown in **Figure** **5**a–c and Figure S11 (Supporting Information), it is not difficult to see that at the 1st Na plating cycle, Na elements are evenly distributed on the N‐CSs substrate consistent with N, indicating that the high content of doped N elements as an effective sodium‐affinity sites guide the uniform deposition of Na. It is well known that the interlayer sodium storage of carbon material generally forms the compound of NaC_8_;^[^ ^25^ ^]^ however, as shown in the C/Na atomic ratio of the delineated area in Figure 5c is about 1.05 that is much higher than the ratio of carbon‐sodium interlayered compounds, proving that the uniformly distributed Na in the Figure exists in the form of Na metal rather than NaC_8_. The TEM and TEM‐EDS mapping images of Na@N‐CSs/Cu at the 300th Na plating cycle were displayed in Figure 5d–f and Figure S12 (Supporting Information). It is noteworthy that after 300th Na plating cycle, N‐CSs have still maintained the layered structure, the uniformly N doping distribution and the even sodium deposition. The C/Na atomic ratio of the region selected in Figure 5f was 0.99 that is similar to the result of Figure 5c, indicating that even after a long cycle, N‐CNs can still effectively inhibit the formation of sodium dendrite and dead sodium. The above results verify the excellent sodium host performance of N‐CSs/Cu.

### 2.4 In Situ XRD and In Situ Sodium Deposition Observation

**Figure 6:**
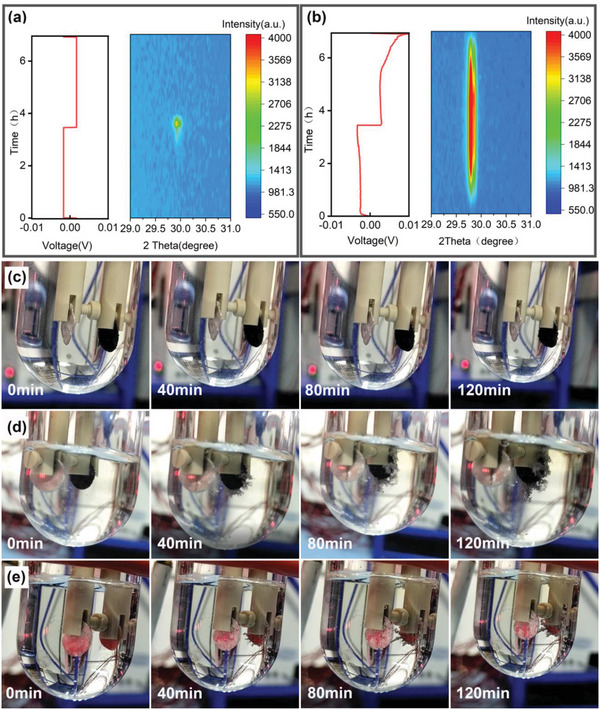
Charge–discharge curve and corresponding in situ XRD patterns of Na deposition on a) N‐CSs, b) N‐CPs. Deposition process of Na metal on different substrates, c) N‐CSs, d) N‐CPs, e) Cu.

In‐situ X‐ray diffraction (XRD) measurements were undertaken to characterize the changes of N‐CSs/Cu and N‐CPs/Cu electrodes during deposition and stripping. In‐situ cells were first discharged to 0 V and then deposited and peeled for 3.5 h at the current density of 1 mA to detect the changes of XRD patterns. As displayed in **Figure** **6**a, during the deposition process, the peak located at 29.8° that is attributed to the signal of Na metal was not observed at N‐CSs/Cu electrode until the deposition last for 3 h, which might be due to the high nitrogen content and wrinkled 2D structural N‐CSs can not only concede dendrite‐free sodium depositing but also accommodate the deposited Na metal in the space between the internal layers rather than aggregation into clusters on the surface of the electrode to be easily detected the XRD signal of sodium metal. As for N‐CPs/Cu electrode, as shown in Figure 6b, the deposition only last for 0.5 h, the characteristic peak of metallic sodium was discovered and the peak intensity increases with the deposition time, which might because that N‐CPs does not have enough space to accommodate the deposited metal sodium so that the deposited metal sodium can only be deposited on the surface of the electrode. Moreover, comparing the XRD patterns of N‐CSs/Cu and N‐CPs/Cu electrodes during the stripping process, we can find that the characteristic peak of metal sodium still exist at the charging cut‐off for N‐CPs/Cu electrodes (Figure 6b), which may be caused by dead sodium, evidencing that the wrinkled 2D structural N‐CSs can not only accommodate sodium depositing but also suppress dead sodium production.

To in situ directly observe the deposition process of Na metal on different substrates, we assembled three liquid‐rich batteries (Figure S13, Supporting Information). At a current density of 1 mA for two hours, we monitored the process with video. As shown in Figure 6, N‐CSs, N‐CPs and bare Cu foils have relatively flat surfaces at the initial stage. During the deposition process for two hours, there was almost no obvious change of N‐CSs/Cu electrode except for the increase in electrode thickness. This evidences that Na metal is deposited in the internal sodium storage space, and the spacing between layers is widened, resulting in volume expansion (Figure 6c; Video S1, Supporting Information). This result explained why the in situ XRD can hardly detect the signal of Na metal. As for N‐CPs/Cu electrode and bare Cu foil, we can find that both electrodes gradually precipitated Na dendrites and grew up during deposition (Figure 6d,e; Videos S2 and S3, Supporting Information). By comparison, it directly shows the superiority of N‐CNs/Cu in sodium dendrite inhibition.

### 2.5 Electrochemical Performance of Practical SMBs

**Figure 7:**
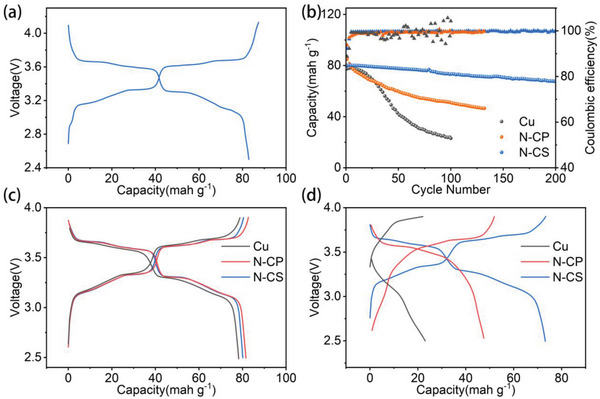
a) Charge–discharge curve of NNMO half‐cell. b) Cycle performance and efficiency comparison of anode‐free cell. Charge–discharge curves comparison at c) 5th, d) 100th.

Finally, the feasibility of N‐CSs, N‐CPs, and Cu anodes in practical sodium metal batteries was explored. A full cell with P2 phase Na_0.67_Ni_0.33_Mn_0.67_O_2_ (NNMO) as cathode was assembled. Figure S14 (Supporting Information) is the XRD curve of NNMO cathode material, which meets the standard PDF card. The charge‐discharge curves of the half‐cell at the current density of 50 mA g^−1^ are shown in **Figure** **7**a, and the specific capacity is 82 mAh g^−1^. As shown in Figure S15 (Supporting Information), the cycling stability of the Na_0.67_Ni_0.33_Mn_0.67_O_2_ cathode has been tested at 200 mA g^−1^. The initial discharge capacity is 82 mAh g^−1^ and the capacity retention rate is about 87% after 300 cycles. We compared the cycle performance of full cell at a current density of 50 mA g^−1^. All three exhibit similar initial specific capacities, yet the capacity retention of N‐CSs|NNMO is 86.3% after 200 cycles with a coulomb efficiency of more than 99.5%, which is much higher than that of N‐CPs|NNMO and Cu|NNMO (Figure 7b). As shown in Figure 7c,d, except for N‐CSs, a large amount of Na was lost due to the deposition/stripping of Na on the anode, resulting in the disappearance of the curve discharge platform and the capacity attenuation. Thus, the N‐CSs substrate exhibits excellent sodium metal affinity and cycling stability.

## 3 Conclusion

In conclusion, N‐CSs/Cu electrode effectively addresses the infinite relative dimension change of sodium metal anodes for SMBs during sodium depositing/stripping and significantly improves the stability of Na metal anodes on account of several unprecedented merits over commonly designed Na metal host anodes. First, the experimental and theoretical calculations show that the high N doping contents of N‐CSs facilitate to reduce sodium nucleation overpotential and guide the uniform and dense sodium metal depositions instead of dendritic growth. Second, the crimped and stacked 2D structural N‐CSs provide sufficient space to accommodate the deposited sodium metal and eliminate the infinite relative dimension change of cycled Na metal anode. Third, N‐CSs can be easily and continuously process into N‐CSs/Cu electrode via traditional commercial battery electrode coating equipment that pave the way for large‐scale industrial applications. Forth, combining the results of ex situ SEM, ex situ TEM, in situ optical video, and in situ XRD, N‐CSs/Cu electrode eliminates the infinite relative dimension change of sodium metal anode during sodium cycling. As a consequence, N‐CSs/Cu electrode demonstrates an superior cycle stability of more than 1500 h at a current density of 2 mA cm^−2^ with a high coulomb efficiency of more than 99.9% and an ultralow nucleation overpotential. As shown in Table S4 (Supporting Information), Several carbon‐based materials in recent years are found as a comparison. This work shows more excellent cycle stability at larger deposition current and capacity. The zero‐volume‐expansion and dendrite‐free N‐CS/Cu electrode provides a practicable and effective cell solution towards high‐performance SMBs and stimulates further large‐scale utilization of SMBs in the future.

## 4 Experimental Section

### Preparation of N‐CSs and N‐CPs

Nitrogen doped carbon sheets (N‐CSs) and nitrogen doped carbon particles (N‐CPs) were prepared by one‐step pyrolysis. The guanine and adenine precursors were both from Sinopharm Chemical Reagent Co. A total of 5 g guanine was placed in a tubular furnace and heated at a heating rate of 3 °C min^−1^ to 800 °C for 3 h in argon atmosphere to obtain N‐CSs. N‐CPs was obtained by treating 5 g adenine with the same method.

### Preparation of Anode and Cathode Electrodes

The anode slurry was prepared by mixing 90% active materials (N‐CSs, N‐CPs) with 10 wt% polyvinylidene fluoride (PVDF) in *N*‐methyl pyrrolidone (NMP). The slurry was uniformly coated on Cu foil with a scraper. Then drying at 105 °C for 24 h. The cathode powder Na_0.67_Ni_0.33_Mn_0.67_O_2_ (NNMO) was synthesized using a previously reported method.^[^ ^26^ ^]^ The active material, Super P and polyvinylidene fluoride (PVDF) were mixed at a mass ratio of 8:1:1 to prepare the slurry, which was coated on Al foil and dried for 24 h. The electrode with a diameter of 8 mm was finally punched. The anode and cathode active material loading were ≈0.4, ≈4 mg cm^−2^.

### Characterizations

Scanning electron microscopy (SEM), transmission electron microscopy (TEM) were performed on TESCAN VEGA3 SBH, Talos F200X FTEM, respectively. X‐ray powder diffraction (XRD), Raman spectroscopy, and X‐ray photoelectron spectroscopy analysis were performed on PANalytical Empyrean diffractometer with Cu K*α* in 10°–60°, Horiba LabRAM‐HR800 employing a 532 nm laser beam, and AXIS‐ULTRA DLD‐600W, respectively.

### Electrochemical Measurements

All electrochemical performance tests were measured in 2032‐type coin cells at room temperature. Complete half‐cell and full cell assembly in argon glove box (O_2_ and H_2_O < 0.1 ppm). The half‐cell used N‐CSs, N‐CPs, bare Cu foil as the working electrode, and Na metal as the reference electrode. 1 m NaPF6 in diglyme was employed as the electrolyte and glass fiber (Whatman) as a separator. 1–10 mAh cm^−2^ Na was deposited on the working electrode to test the cycle stability and rate performance. The cut‐off voltage is 0.3 V. The full cell was assembled by NNMO cathode and N‐CS, N‐CP, Cu anode. The anode and cathode active material loading were ≈0.4, ≈4 mg cm^−2^. All three anodes were predeposited with 0.5 mAh Na. 1 m NaPF6 in diglyme was employed as the electrolyte and glass fiber (Whatman) as a separator. The above electrochemical tests were carried out on LAND CT2001A at 2.5–3.9 V. Electrochemical impedance spectroscopy (EIS) tests were carried out in the frequency range of 0.1–100 000 Hz.

### Computational Details

All calculations were performed by projector augmented‐wave (PAW) method and implemented in Vienna ab initio simulation package (VASP) code.^[^ ^27^, ^28^ ^]^ The exchange‐correlation interaction was calculated by generalized gradient approximation (GGA) of Perdew–Burke–Ernzerhof (PBE).^[^ ^29^ ^]^ A kinetic energy cutoff of 500 eV was used for the expansion of the Kohn–Sham orbitals in plane waves. The optimize precision of total energy difference was 10^−6^ eV for all the structures and the Hellman–Feynman forces on each atom converged to 0.01 eV Å^−1^. The *k*‐point sampling and Brillouin zone integration were operated with Monkhorst–Pack scheme^[^ ^30^ ^]^ of 2×2×1 grid points for the calculations of adsorption energies.

To calculate the adsorption energies of sodium atom adsorbed on graphene, N doped graphene and Cu (001) plane, the following equation is performed

(1) $$E_{\mathrm{ads}}=E_{\left(\mathrm{Na}+\mathrm{slab}\right)}-E_{\left(\mathrm{slab}\right)}-E_{\left(\mathrm{Na}\right)}$$

Where *E*_ads_ is adsorption energy; *E*_(Na+slab)_, *E*_(slab)_, *E*_(Na)_ are total energy of Na atom adsorbed on slab, slab model, and the energy per sodium atom in sodium metal, respectively.

## Conflict of Interest

The authors declare no conflict of interest.

## Supporting information

Supporting Information

Supplemental Video 1

Supplemental Video 2

Supplemental Video 3

## Data Availability

Research data are not shared.
