# Supplementary material for: Ultrahigh Nitrogen Content Carbon Nanosheets for High Stable Sodium Metal Anodes
Source: Adv Sci (Weinh). 2023 Feb 15;10(11):2206845. doi: 10.1002/advs.202206845 (PMC10104674; doi:10.1002/advs.202206845)
Supplement: Supplementary file 1 — Supporting Information [file ADVS-10-2206845-s003.pdf]

Supporting Information

Ultrahigh nitrogen content carbon nanosheets for high stable sodium metal anodes

Bicheng Huang,[†a] Shixiong Sun,[†*a] Jing Wan,[b] Wen Zhang,[a] Siying Liu,[a] Jingwen Zhang,[a] Feiyang Yan,[a] Yi Liu,[a] Jia Xu,[a] Fangyuan Cheng,[a], Yue Xu, [a] Yaqing Lin,[*a] Chun Fang, [*a] Jiantao Han,[a] Yunhui Huang [a]

[a] State Key Laboratory of Material Processing and Die & Mould Technology, School of Materials Science and Engineering, Huazhong University of Science and Technology, Wuhan 430074, P. R. China

[b] Department of Applied Physics, Chongqing University, Chongqing 401331, China.

[*] Corresponding author. Tel. & fax: 86-27-87558241.

E-mail:fangchun@hust.edu.cn (C. Fang), shx19870502@126.com (S.Sun)

Sample	BET surface area (m^2g^{-1})	Pore Volume (cm^3g^{-1})	Pore diameter (nm)
N-CSs	76.54	0.2006	12.24
N-CPs	4.476	0.0043	13.27

Table S1. Textural property of N-CSs and N-CPs.

Sample	C(at.%)	N(at.%)	O(at.%)
N-CSs	69.12	27.03	3.85
N-CPs	70.44	26.63	2.93

Table S2. C, N, O atom content of N-CSs and N-CPs from TEM-EDS mapping.

Sample	C(at.%)	N(at.%)	O(at.%)	N-5(at.%)	N-6(at.%)	N-Q(at.%)
N-CSs	62.40	33.40	4.20	6.30	14.20	12.90
N-CPs	71.54	25.08	3.38	9.84	11.34	3.9

Table S3. C, N, O atom content of N-CSs and N-CPs from XPS.

Host/Na	electrolyte	current density ($mA\ cm^{-2}$)	stripping capacity ($mAh\ cm^{-2}$)	overpotential (mV) (cycles)
r-GO/Na	1 M NaPF6-diglyme	1	1	~20 (300)
carbon felt/Na	1 M NaClO4-EC/PC	5	2	~100 (125)
carbonized wood/Na	1 M NaClO4-EC/DEC	1	1	>200(250)

carbon fiber/Na	1 M CF3SO3Na-diglyme	1	1	~20 (500)
This work	1 M NaPF6-diglyme	2	2	~15(700)

Table S4. Comparison of Electrochemical Performance of Carbon-based Materials

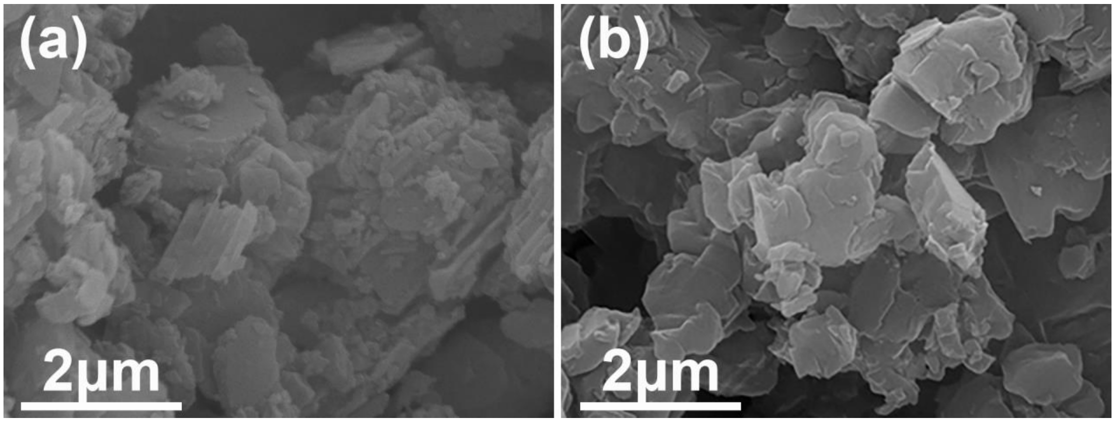

Figure S1. a)SEM image of guanine. b)SEM image of adenine.

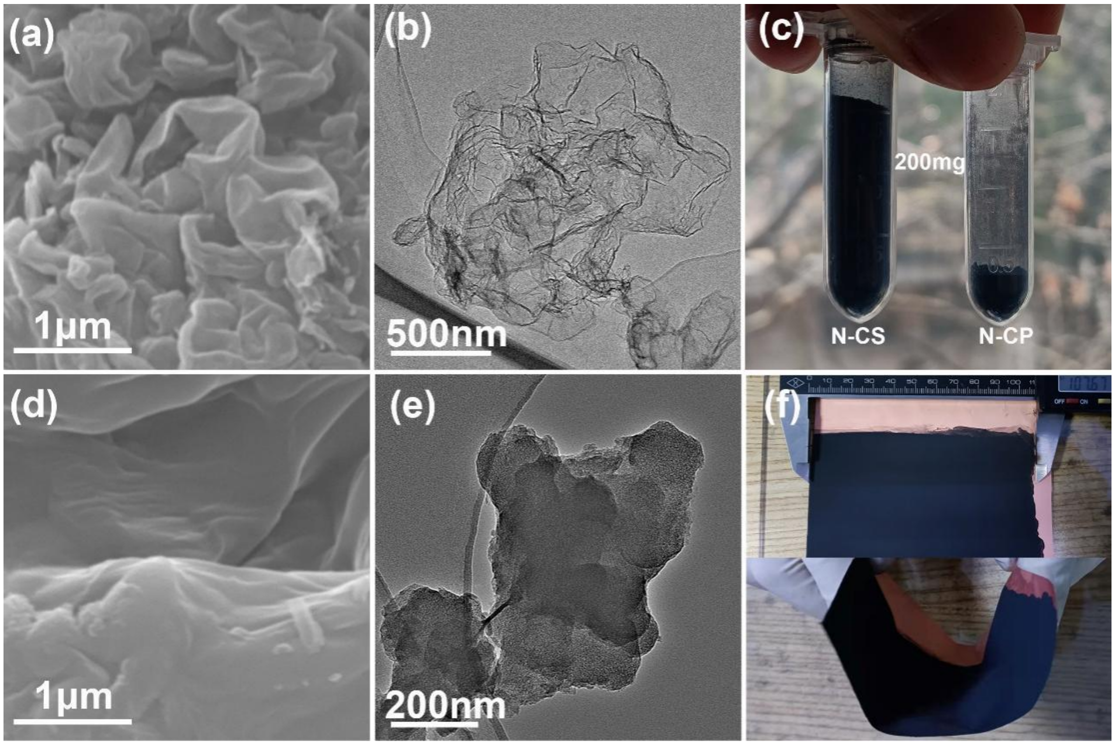

Figure S2. a,d) SEM image of N-CSs and N-CPs. b,e) TEM image of N-CSs and N-CPs. c) Optical pictures of N-CSs and N-CPs. f) Electrode slides of N-CSs.

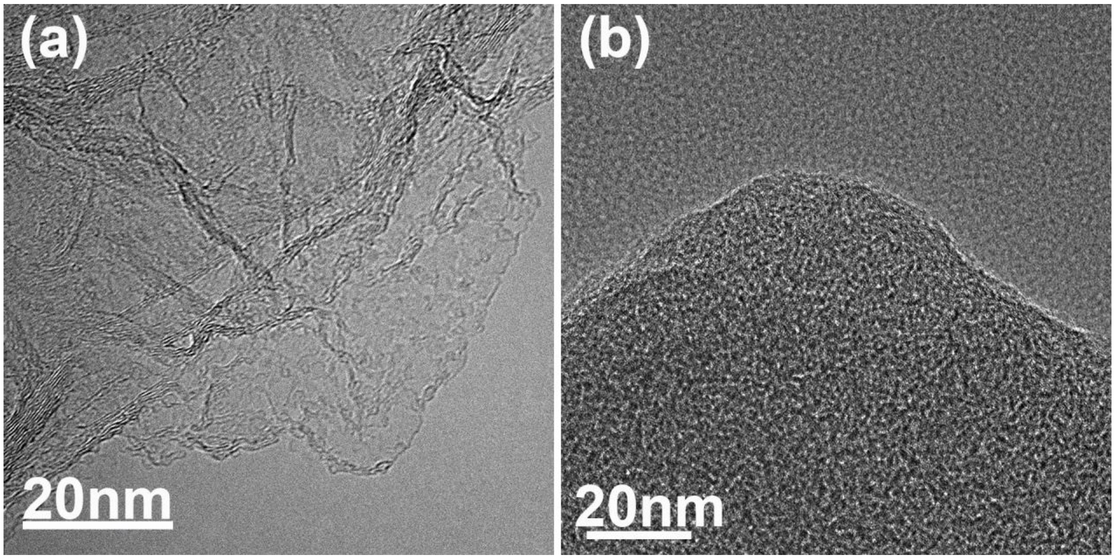

Figure S3. a)HRTEM image of N-CSs. b)HRTEM image of N-CPs.

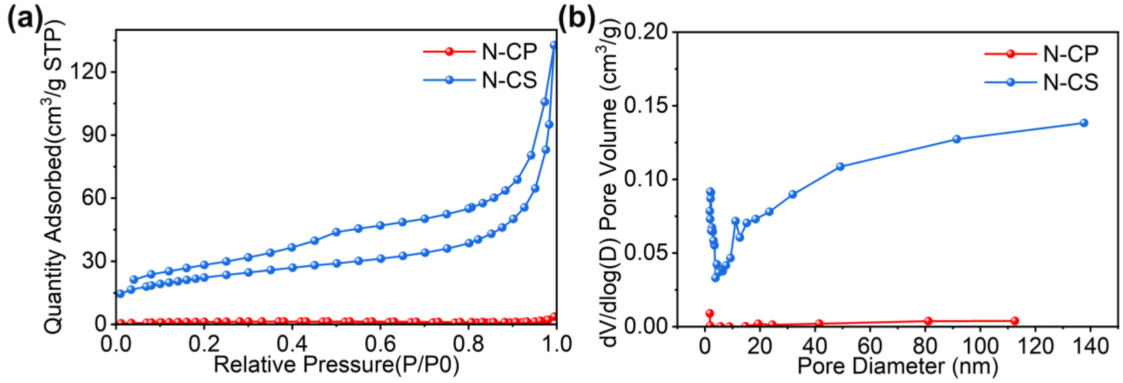

Figure S4. a)Brunauer-Emmett-Teller (BET) nitrogen adsorption/desorption isotherms of N-CSs and N-CPs. b)BJH pore size distribution profiles of N-CSs and N-CPs.

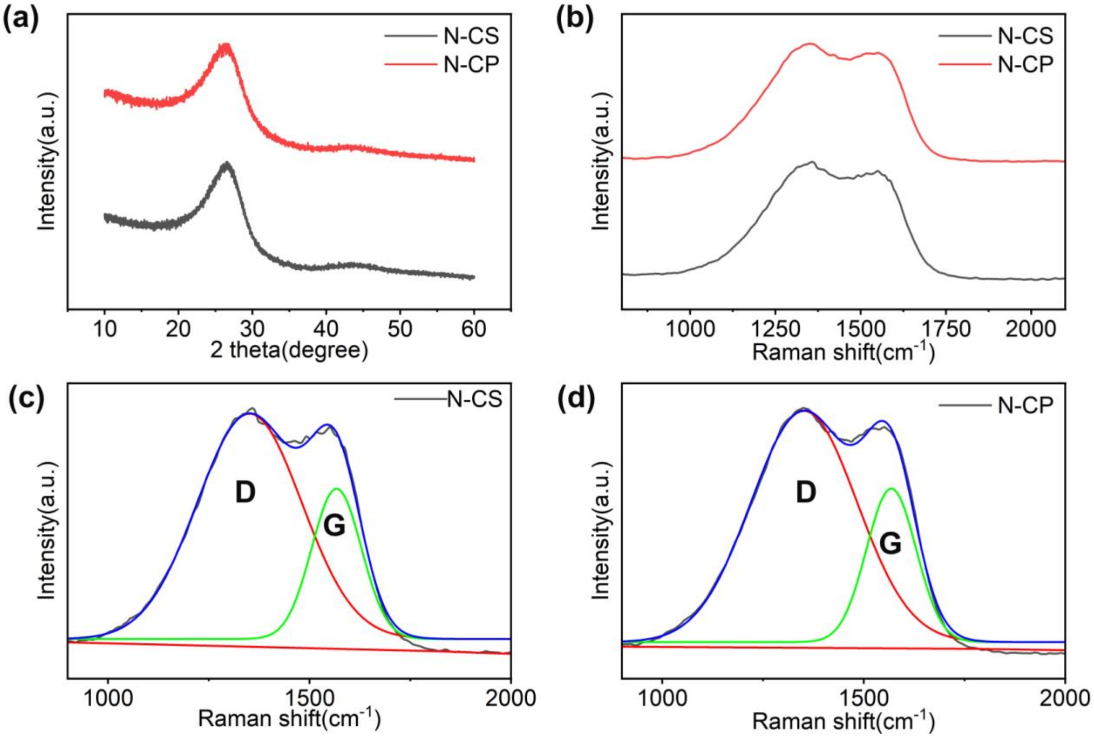

Figure S5. a) XRD data of N-CSs and N-CPs. b) Raman spectrum of N-CSs and N-CPs. c) Raman spectrum peak fitting of N-CSs. d) Raman spectrum peak fitting of N-CPs.

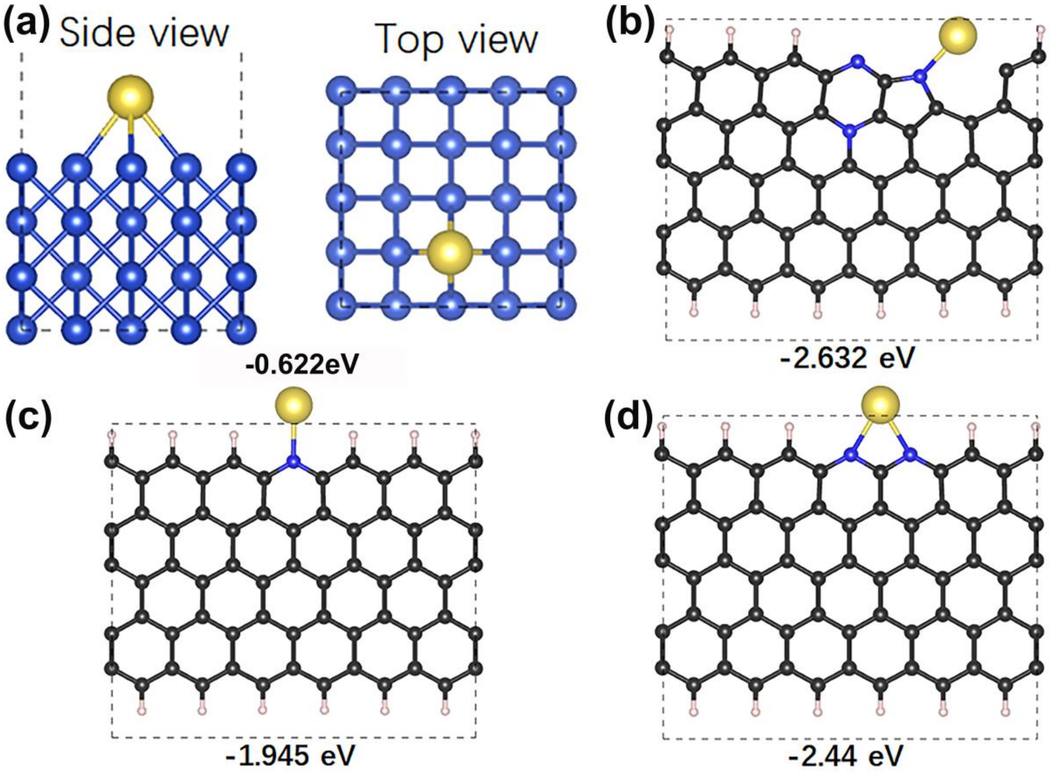

Figure S6. Computational models of a) Cu, b)N-5, N-6, N-Q co-doping, c) Single N-6 doping and d) Double N-6 doping.

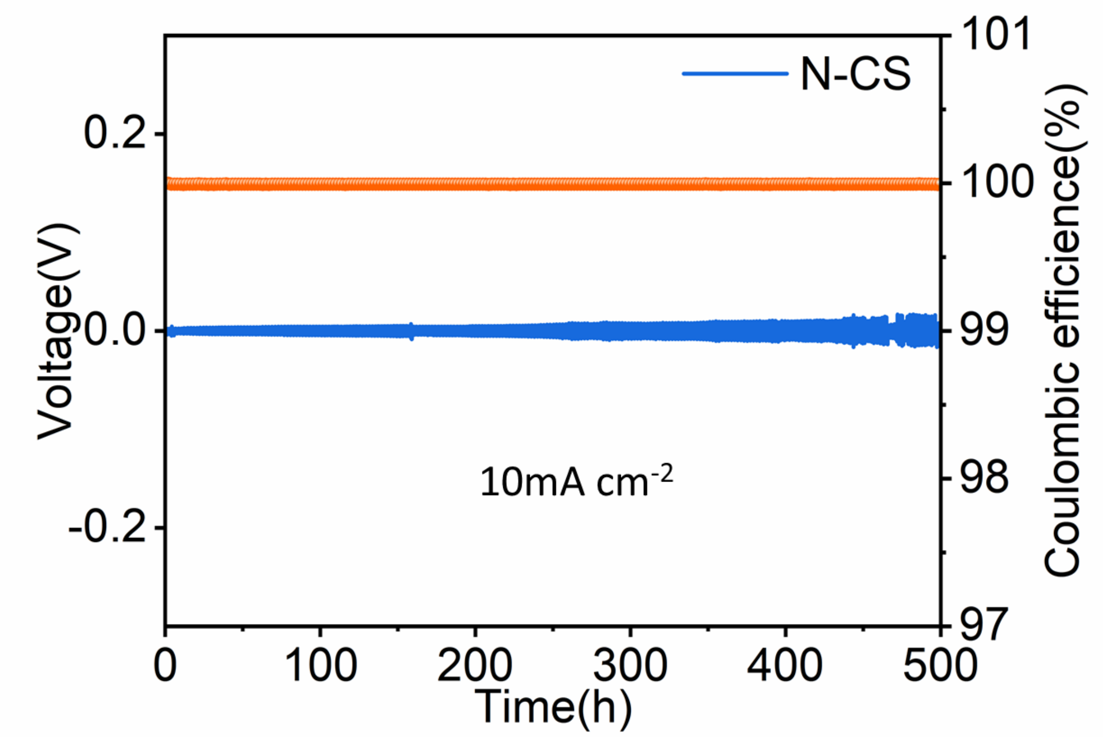

Figure S7. High Current Cycle Stability of N-CSs at 10mA cm^{-1}.

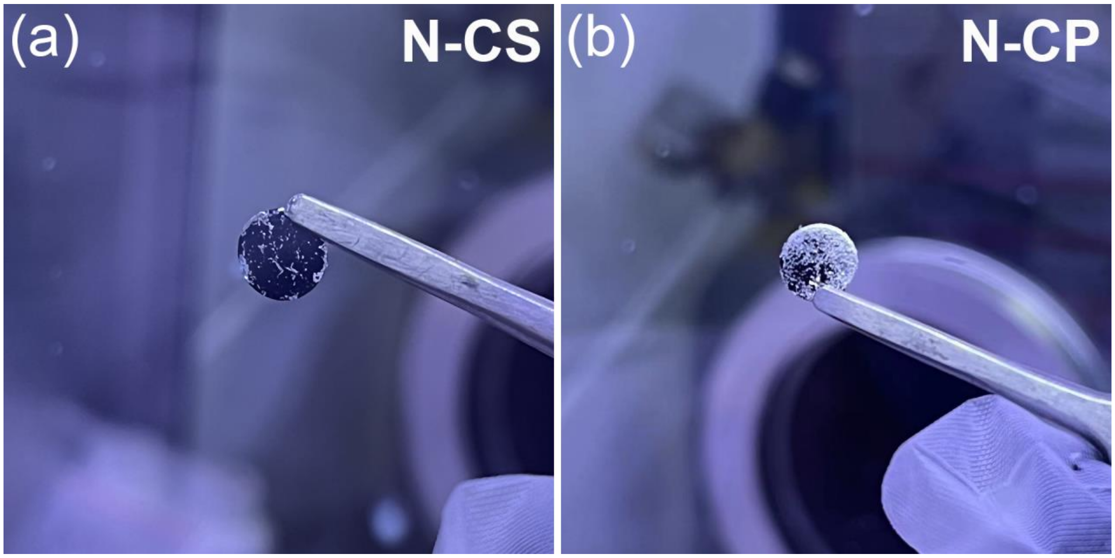

Figure S8. Optical photographs of plated sodium metal on a) N-CSs, b) N-CPs.

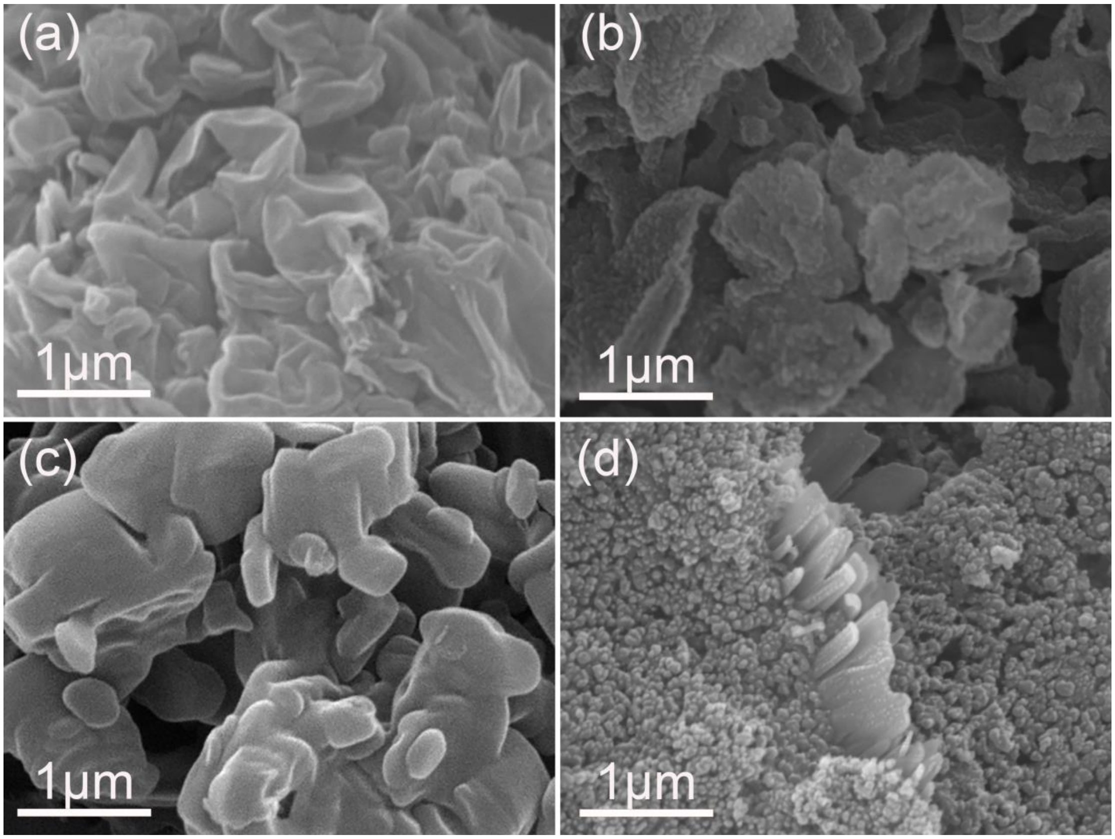

Figure S9. High magnification SEM images of a) N-CSs/Cu, b) plated Na@N-CSs/Cu, c)N-CPs/Cu, d) plated Na@N-CPs/Cu.

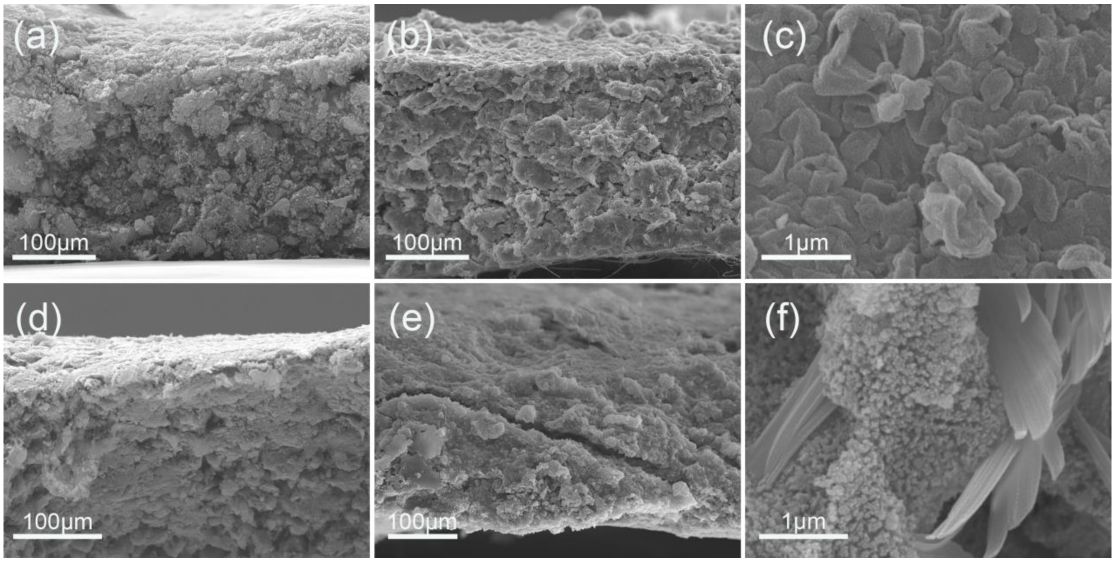

Figure S10. N-CSs/Cu Cross-sectional SEM images of a) before plating, b) after plating, c) high magnification after plating. N-CPs/Cu Cross-sectional SEM images of d) before plating, e) after plating, f) high magnification after plating.

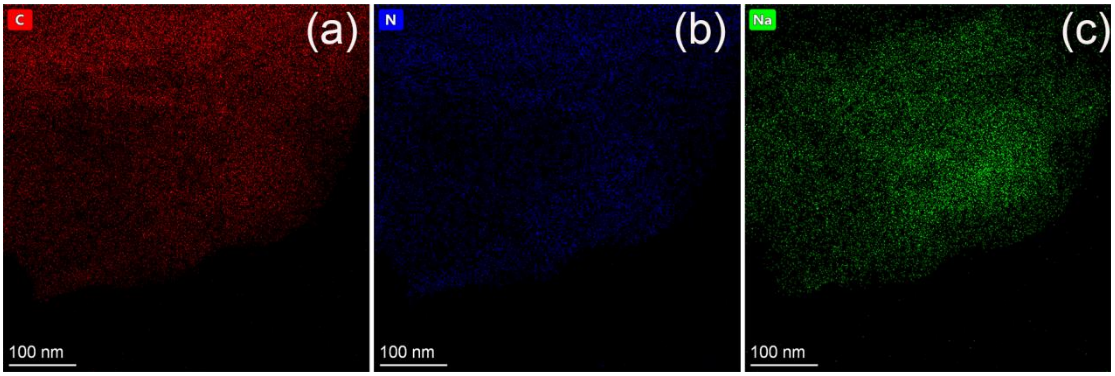

Figure S11. TEM-EDS mapping images of Na@N-CSs/Cu after Initial deposition a) C, b) N, c) Na.

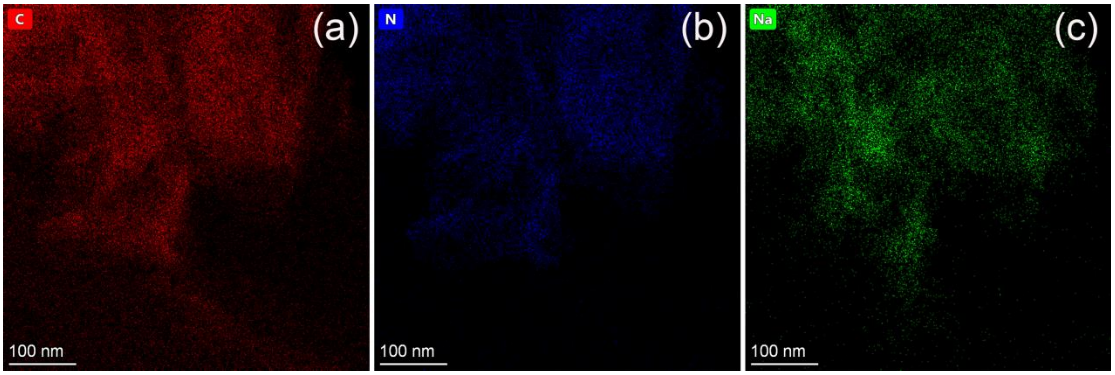

Figure S12. TEM-EDS mapping images of Na@N-CS@Cu after 300 cycle a) C, b) N, c) Na.

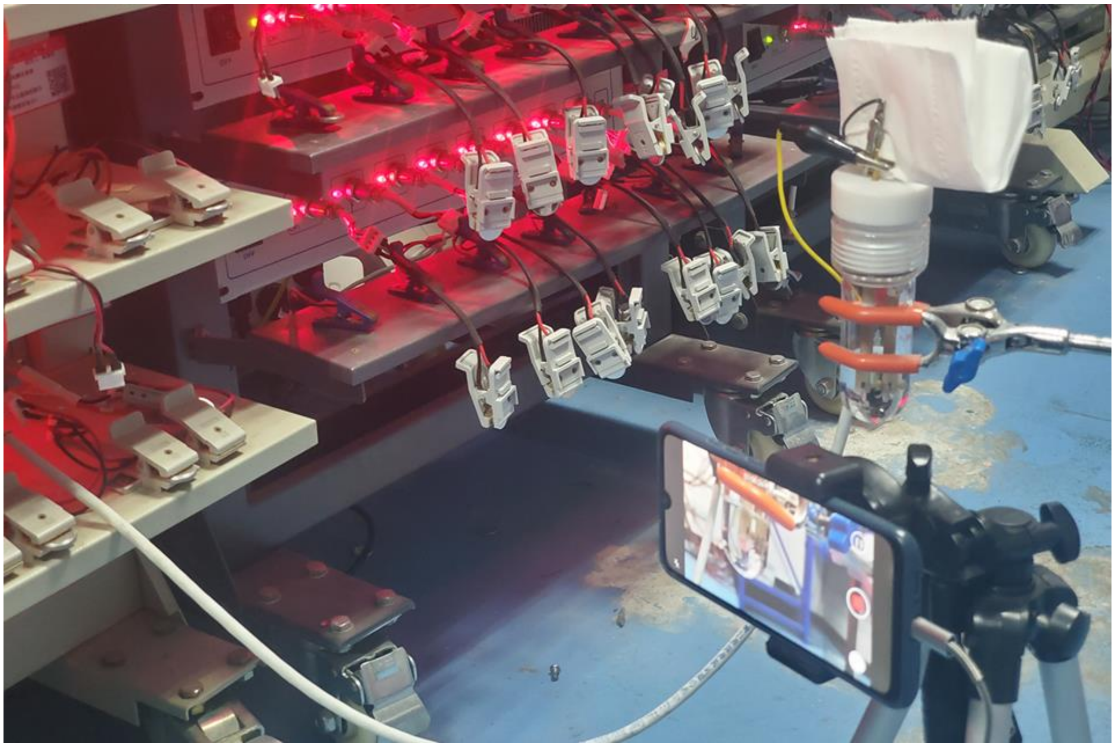

Figure S13. In-situ photographic equipment of liquid-rich batteries.

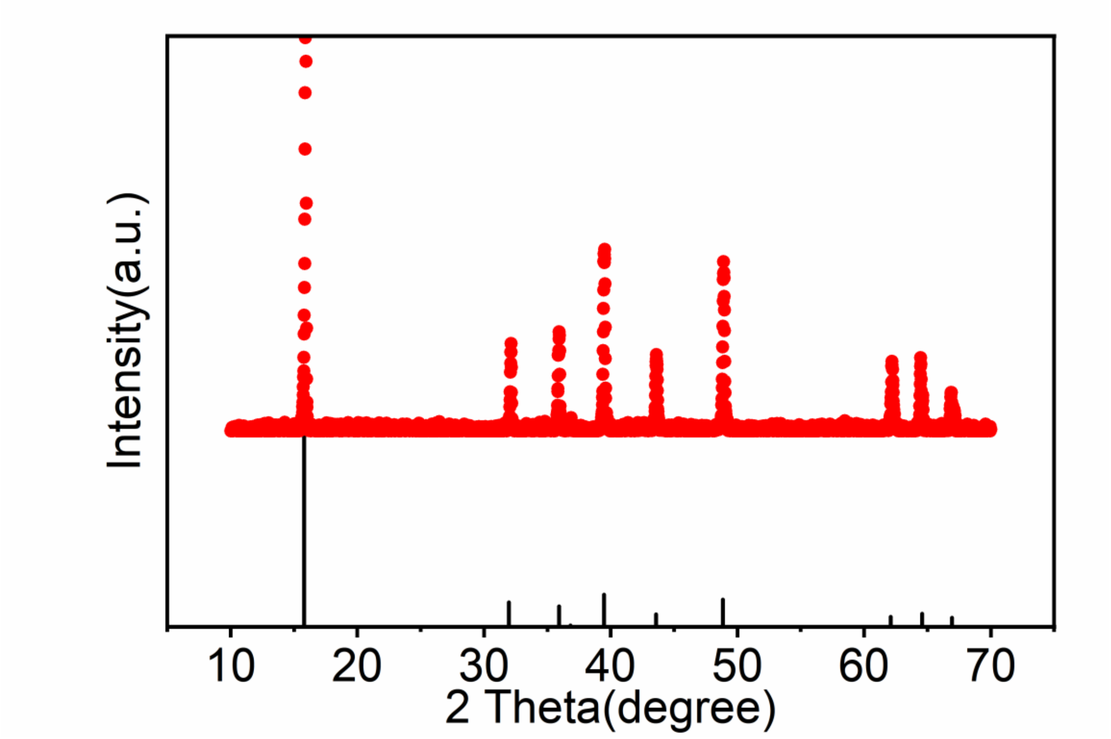

Figure S14. XRD patterns of $Na_{0.67}Ni_{0.33}Mn_{0.67}O_2$ (PDF standard card 70-3726)

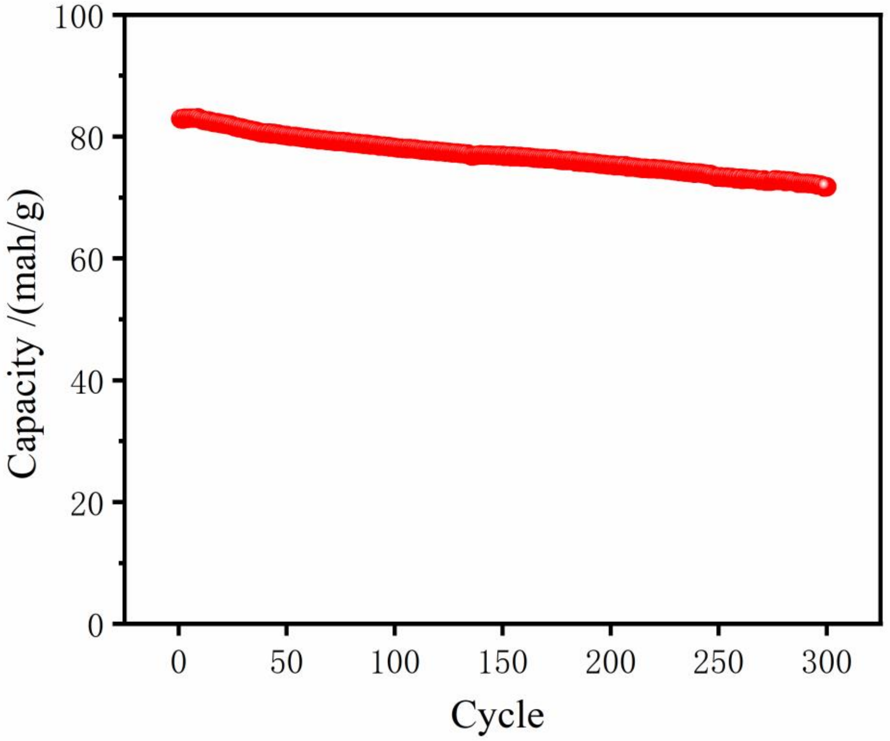

Figure S15. The cycling stability of the $Na_{0.67}Ni_{0.33}Mn_{0.67}O_2$
